# Reproduction and community structure of fish from winter catch sites from industrial shrimp bycatch from the northeast and southeast Mexican Pacific

**DOI:** 10.7717/peerj.4460

**Published:** 2018-03-02

**Authors:** Jorge de Jesus Tirado-Ibarra, Mariany Loya-Rodriguez, Jose Carlos Morales-Arevalo, Isabel Rosario Muñoz-Garcia, Francisco Martinez-Perez, Jorge Saul Ramirez-Perez, Laura Rebeca Jimenez-Gutierrez

**Affiliations:** 1 Facultad de Ciencias del Mar, Universidad Autonoma de Sinaloa, Mazatlan, Sinaloa, Mexico; 2 Laboratorio de Genómica de Celomados del Grupo de Microbiología y Genética, Universidad Industrial de Santander, Bucaramanga, Santander, Colombia; 3 CONACyT, Direccion de Catedras-CONACYT, Ciudad de Mexico, Mexico

**Keywords:** Community structure, Length of maturity, Finfish, Shrimp bycatch

## Abstract

The shrimp fishery is one of the most important fisheries in the world, although the low selectivity from trawling nets has led to the capture of a large number of non-target species. Shrimp-bycatch species include a large number of fish and invertebrate species, of which fish species are the most abundant. The present study aims to determine the community structure as well as the average sizes at first maturity of the fish species from shrimp-bycatch caught from industrial fisheries in the Mexican Pacific from Sinaloa to Guerrero, from January to March 2015. The shrimp-bycatch fish diversity value was found to be 2.22. A total of 37 species of finfish were found, of which five were considered rare. The fish species with the highest Importance Value Index (IVI) levels were *Pseudupeneus grandisquamis*,* Paralichthys woolmani*,* Lutjanus peru* and *Diapterus peruvianus*. The average size at first maturity was calculated for all species. Of the analysed organisms, 90% were in the juvenile stage, including species with riverine and artisanal fisheries. The present study demonstrates the risk within marine populations to different non-target species due to the poor selectivity of shrimp trawls.

## Introduction

Shrimp, which are among the most traded fishery products in the world, generate numerous important economic benefits (FAO, 2017), in both riverine and industrial fishery. As with other fisheries, shrimp fishery is not perfect (Clucas, 1997), especially industrial fishery. Recent years have seen an on-going emphasis on the impact of this activity on shrimp-bycatch fauna; the FAO considers this fishery the main source of discarded species (Kelleher, 2004; Kumar & Deepthi, 2006; FAO, 2017). The Gulf of California, widely considered the ‘world’s aquarium’ because of its biological diversity (Enríquez-Andrade et al., 2005) and the Mexican Pacific are the most important bodies of water for the Mexican shrimp fishery (Arvizu-Martinez, 1987; Magallon-Barajas, 1987).

Because shrimp are benthonic organisms, shrimp-bycatch includes a great diversity of fauna, including molluscs, echinoderms, crustaceans and various fishes. The most abundant species among shrimp-bycatch fauna are fishes, including more than 100 small-size fish species (Perez-Mellado & Findley, 1985) such as flatfishes, snappers and pufferfishes, among others (Aguirre et al., 2008; Gibinkumar et al., 2012; Rábago-Quiroz et al., 2011). More than 114,000 tons of discarded fish are generated per year (Bojorquez, 1998).

Even the term ‘bycatch’ has many definitions and considerations. Shrimp-bycatch comprises tons of non-target organism catch due to the poor selectivity of the trawls; these species are often discarded by fishery vessels or become destined for local consumption (Alverson et al., 1994). The main concern, however, is not only to search for potential uses of bycatch (Kelleher, 2004), but also to implement more selective trawls in order to reduce bycatch to a minimum. The poor selectivity of the trawls damages entire ecosystems and could deplete populations (Alverson et al., 1994), thus affecting even target species.

Tropical shrimp trawl fisheries are often small and have little or no room for bycatch, which means that bycatch is not cost-effective; this is the main reason that bycatch is discarded (Kelleher, 2004). At the global level, on-going trawl design innovations (Boopendranath et al., 2013) have led to greater protection of sea turtles and sea mammals (Norma Oficial Mexicana, 1996; Morzaria-Luna et al., 2013; Norma Oficial Mexicana, 2013); which, according to the Official Mexican Norm are threatened (Norma Oficial Mexicana, 2010). Still, less charismatic and less economically important species die either during or after trawling (Hill & Wassenberg, 1990; Ramírez-Ramírez et al., 2008). These organisms are then returned to the sea, which leads to environmental contamination (Ramírez-Ramírez et al., 2008).

In tropical countries each year, shrimp-bycatch corresponds to more than 90% of the catch (Alverson et al., 1994; Kelleher, 2004). Some of these organisms have greater marketing potential. Still, the various biological aspects of shrimp-bycatch fish are not well understood (Ramos-Santiago et al., 2006; Herrera-Valdivia, López-Martínez & Morales-Azpeitia, 2016). Because population studies of low-commercial-importance species are not cost-effective, bycatch studies are an excellent opportunity to examine these seldom-studied species (Foster & Vincent, 2010).

Shrimp-bycatch species must be evaluated not only because of their importance to their ecosystems and their potential commercial value (Clucas, 1997; Pauly et al., 1998; Foster & Vincent, 2012) but also because it is important to learn their size structure and reproductive status. Most shrimp-bycatch fauna are under 20 cm (the same size as tropical adult shrimp) and weigh less than 100 g (Alverson et al., 1994; Foster & Arreguin-Sánchez, 2014); this is because smaller organisms must work harder to escape, so many of them die from exhaustion in the trawl (Liggins & Kennelly, 1996). Most of these organisms are juveniles or sub-adults, most of which have not yet reproduced at least once (Hilborn & Walters, 1991). These factors all significantly affect the recruitment of future generations (Broadhurst, 2000).

In addition, the lack of information about the size at first maturity and the reproductive periods, as well as the minimum sizes of catches of less commercially valuable species, have all led to an absence of regulations for small fisheries (Foster & Vincent, 2010; Morzaria-Luna et al., 2013). The aim of this work is thus to determine the population structure and relative abundance of the fish species present in shrimp-bycatch from industrial shrimp fishery the northeast and southeast Mexican Pacific, as well as their spatial variability and potential risk involved in the recruitment of the species. Such information will serve as the basis for determining the actual status of marine communities and the effect of human activities in each region.

## Materials and Methods

**Figure 1:**
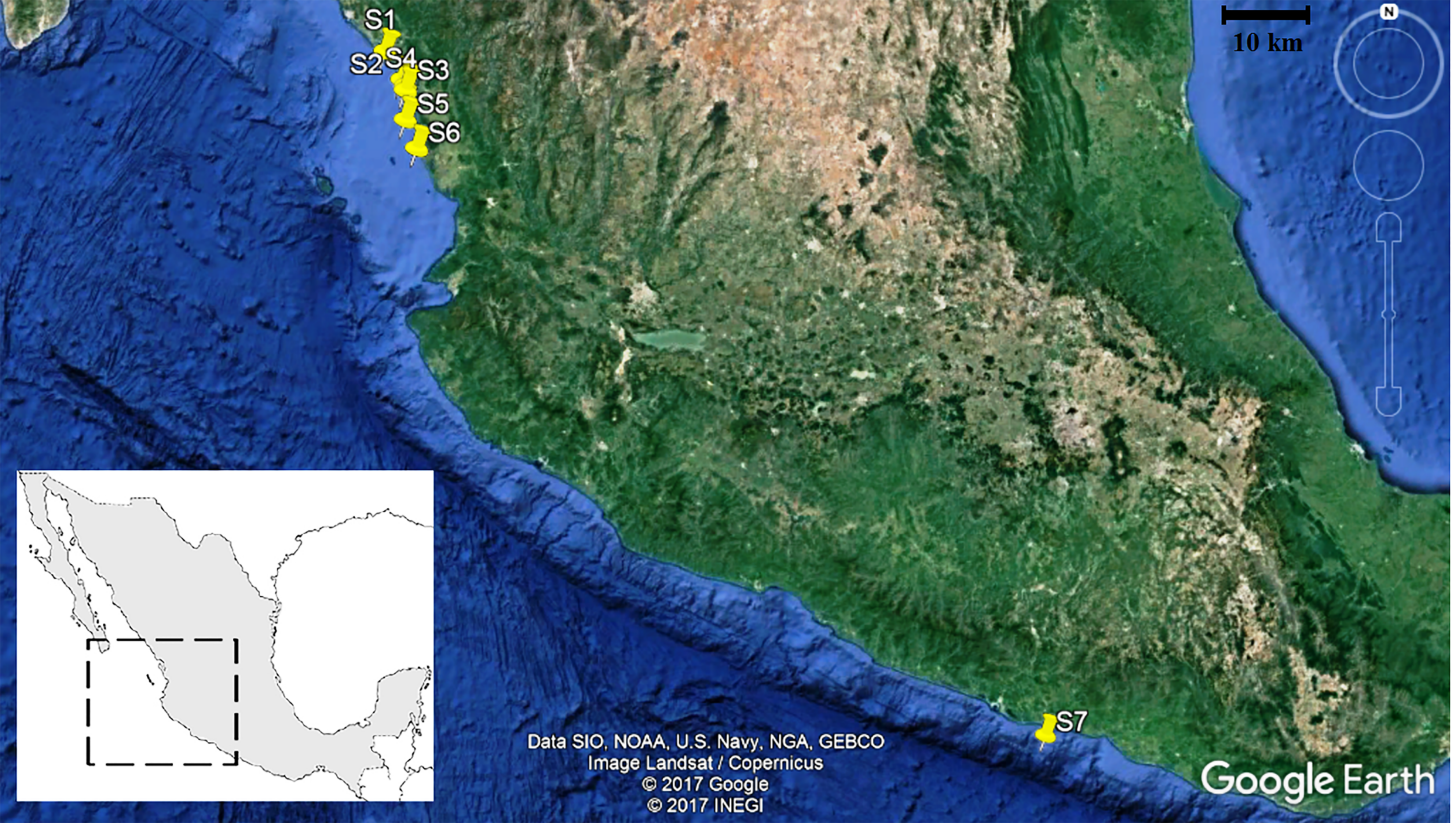
Location of the seven shrimp catches stations in the northeast and southeast of the Mexican Pacific. The square within the map of Mexico indicates the satellite image. Each station is indicated in yellow. Black bar represents 10 km. Map data © Google, INEGI, Data SIO, NOAA, U.S. Navu, NGA, GEBCO, Image Landsat/Copernicus.

This study is based on an analysis of bycatch obtained from an industrial shrimping vessel’s trawling while operating on the Mexican continental shelf from Sinaloa to Guerrero (to this work this geographical area is considered Guerrero as the southeast from the Mexican Pacific and Sinaloa as northeast of the Mexican Pacific and the southeast from the Gulf of California). The work included the fauna obtained from seven catch sites (from 16.40722 to 24.40010 N and 99.42970 to 108.10895 W; Fig. 1). The catches were made during the second half of the fishing period, from January 26 to March 20, 2015, using a 70-foot-long (21.3-m) trawl with a 38-mm net mesh. Each trawl was submerged for two hours at approximate depths of 20 and 54 m, according to Norma Oficial Mexicana (2013).

After the target species (shrimp) was separated on the vessel, a random 10-kg sample from the bycatch fauna was taken, from which all finfish species were separated and identified at the species level (Allen & Robertson, 1994). The Total Length (TL) of each organism was then measured, and gonadal maturity and sex were analysed using morpho-colorimetric methods (Bucholtz, Tomkiewicz & Dalskov, 2008; Sánchez et al., 2013); the sex was only determined in organisms with developed gonads.

### Community structure

Total abundance and abundance per station of each species were then estimated. The Shannon-Weaver diversity index (*H*′) was calculated at each station:

\begin{eqnarray*}& & {\mathbi{H}}^{{^{\prime}}}=-\sum \mathrm{P}i \mathrm{ln} \mathrm{P}i \end{eqnarray*}

\begin{eqnarray*}& & \mathbf{P}\mathbi{i}=ni/N. \end{eqnarray*}

The similitude at each pair of stations and each pair of sampling months was calculated by the Morisita similitude index (*I*; Morisita, 1959), with modifications, in order to allow for interpretation of the results as a percentage of similitude among samples:

\begin{eqnarray*}& & \mathbi{I}= \frac{2\sum Xi Yi}{(\lambda 1+\lambda 2)(N1 N2)} \times 100 \end{eqnarray*}

\begin{eqnarray*}& & \lambda \mathbf{1}= \frac{\sum [Xi(Xi-1)]}{N1(N1-1)} \end{eqnarray*}

\begin{eqnarray*}& & \lambda \mathbf{2}= \frac{\sum [Yi(Yi-1)]}{N2(N2-1)} . \end{eqnarray*}

In addition, the Relative Density (RD), Relative Frequency (RF) and Importance Value Index (IVI; Smith & Smith, 2006) were calculated for each species. IVI values indicated the degree of dominance of each species and their degree of constancy within the ecosystem:

\begin{eqnarray*}& & \mathbf{RD}= \frac{\mathrm{Total~ number~ of~ individuals~ of~ a~ species}}{\mathrm{Total~ number~ of~ individuals~ of~ all~ species}} \times 100 \end{eqnarray*}

\begin{eqnarray*}& & \mathbf{RF}= \frac{\mathrm{Frequency~ of~ one~ species}}{\mathrm{Total~ frequency~ of~ all~ species}} \times 100 \end{eqnarray*}

\begin{eqnarray*}& & \mathbf{IV I}=\mathrm{RD}+\mathrm{FR}. \end{eqnarray*}

The 12 fish species with the highest IVI were then selected for an evaluation of the size structure per station. The sizes of the organisms were analysed to determine the normality and homoscedasticity of the sample. A one-way ANOVA at α < 0.05 was used to find any significance among the data. Finally, the differences among stations were calculated via Tukey-Kramer multiple comparison tests with a 95% confidence level using the NCSS 2007 statistical programme.

### Length at maturity (*TL*_*m*_)

In order to facilitate estimation of *L*_*m*_ in the absence of suitable data, an empirical relationship based on linking *L*_*m*_ with *L*_∞_was used, as proposed by Froese & Binohlan (2000). The resulting values were compared with the size structure among the sampled stations:

\begin{eqnarray*}& & \mathbf{log} {\mathbi{L}}_{\infty }=0.044+0.9841\ast \mathrm{log}(T{L}_{\mathrm{max}}). \end{eqnarray*}

\begin{eqnarray*}& & {\mathbi{TL}}_{\mathbi{m}}=0.8979\ast \mathrm{log}({L}_{\infty })-0.0782. \end{eqnarray*}

*TL*_max_ was then obtained for each species in the sample from FishBase.org.

## Results

### Community structure

All fish species analysed in this study belong to the superclass Osteichthyes. The specific richness (S) was found to be among 13 and 17 species (Table 1). The total abundance of the study was 1,414 fish from 37 species, belonging to 28 families and 35 genera. The global Shannon-Weaver diversity value was found to be 2.22, with the highest diversity at station 3 (2.118). The stations’ similitudes were among 39% and 93%, with the highest similitude among stations 1 and 7, which were the most geographically distant stations. Otherwise, the month similitudes were among 75% and 83%.

**Table 1:** Species richness, Shannon-Weaver diversity and Morisita similitude from shrimp-bycatch species from the northeast and southeast Mexican Pacific. (A) Species richness and Shannon-Weaver diversity at each station. (B) Morisita similitude index from each peer of stations. (C) Morisita similitude index from each peer of sampled months. J, January; F, February; M, March.

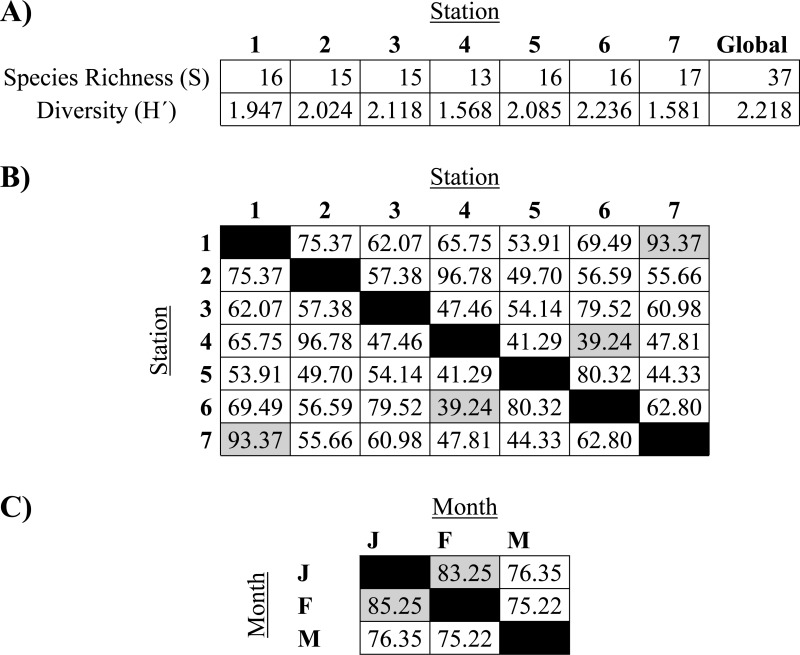

**Table 2:** Fishes IVI from shrimp bycatch from the northeast and southeast Mexican Pacific.

Family	Specie	Freq	RD	RF	IVI	*TL*_**max**_ (cm)	*TL*_***m***_ (cm)
Mullidae	*Pseudupeneus grandisquamis*	HF	28.15	100	128.15	30	18.5
Paralichthyidae	*Paralichthys woolmani*	HF	22.63	100	122.63	80	44
Lutjanidae	*Lutjanus peru*	HF	15.06	100	115.06	95	51.2
Gerreidae	*Diapterus peruvianus*	HF	8.91	100	108.91	30	18.5
Tetraodontidae	*Sphoeroides pachygaster*	HF	5.16	85.71	90.87	40.5 SL	24.1 SL
Haemulidae	*Pomadasys panamensis*	HF	4.81	85.71	90.52	39	23.3
Carangidae	*Chloroscombrus orqueta*	HF	1.13	85.7	86.83	30	18.5
Synodontidae	*Synodus scituliceps*	F	2.26	71.42	73.68	42	24.9
Triglidae	*Prionotus horrens*	F	0.85	71.42	72.27	35	21.2
Fistulariidae	*Fistularia corneta*	F	0.78	71.42	72.2	106	56.3
Serranidae	*Diplectrum macropoma*	F	2.83	57.14	59.97	18	11.7
Achiridae	*Achirus mazatlanus*	F	0.28	57.14	57.42	20	12.9
Batrachoididae	*Nautopaedium porosissimum*	F	0.24	57.14	57.42	32	19.5
Ophichthidae	*Ophichthus triserialis*	F	0.28	42.85	43.13	122	63.8
Balistidae	*Balistes polylepis*	LF	0.21	42.85	43.06	76	42
Sciaenidae	*Larimus argenteus*	LF	0.21	42.85	43.06	35	21.2
Carangidae	*Selene brevoortii*	LF	0.85	28.57	29.42	38 FL	22.8 FL
Ophidiidae	*Lepophidium prorates*	LF	0.42	28.57	28.99	29.5 SL	18.2 SL
Sciaenidae	*Isopisthus remifer*	LF	0.35	28.57	28.92	36	21.7
Clupeidae	*Opisthonema libertate*	LF	0.28	28.57	28.85	30	18.5
Carangidae	*Caranx otrynter*	LF	0.21	28.57	28.78	60	34.1
Bothidae	*Bothus constellatus*	LF	0.14	28.57	28.71	15.7	10.4
Cynoglossidae	*Symphurus prolatinaris*	LF	0.14	28.57	28.71	16.1 SL	10.6 SL
Carangidae	*Trachinotus kennedyi*	LF	0.14	28.57	28.71	90	48.8
Tetraodontidae	*Sphoeroides testudineus*	LF	1.27	14.28	15.55	38.8	23.2
Polynemidae	*Polydactylus opercularis*	LF	0.92	14.28	15.2	50	29
Scorpaenidae	*Scorpaena sonorae*	LF	0.42	14.28	14.7	15.8 SL	10.5 SL
Tetraodontidae	*Sphoeroides annulatus*	LF	0.21	14.28	14.49	44	25.9
Chaetodontidae	*Chaetodon humeralis*	LF	0.21	14.28	14.49	25.4	15.9
Engraulidae	*Anchoa walkeri*	LF	0.21	14.28	14.49	14.5	9.7
Gobiidae	*Bollmannia stigmatura*	LF	0.14	14.28	14.42	14 SL	9.4
Haemulidae	*Xenichthys xanti*	LF	0.14	14.28	14.42	24	15.2
Ariidae	*Bagre pinnimaculatus*	R	0.07	14.28	14.35	95	51.2
Sciaenidae	*Bairdiella armata*	R	0.07	14.28	14.35	30	18.5
Bythitidae	*Brotula clarkae*	R	0.07	14.28	14.35	115	60.6
Paralichthyidae	*Ancylopsetta dendritica*	R	0.07	14.28	14.35	35	21.2
Sphyraenidae	*Sphyraena guachancho*	R	0.07	14.28	14.35	200	98

**Notes.**

RD Relative Density RF Relative Frequency IVI Importance Value Index; from highest to lowest IVI Freq Frequency R Rare LF Low frequent F Frequent HF High Frequent *TL*_max_ Maximum length (FishBase.org) *TL*_*m*_ Length of maturity SL Standard length FL Fork length

To this research, species with one individual (i.e., it only appeared in one station) were considered rare; these species corresponded to 16% of fish species (Table 2). The rare species were *Bagre pinnimaculatus*, *Bairdiella ronchus*, *Brotula clarkae*, *Ancylopsetta dendrítica* and *Sphyraena guachancho*. Only 13 species had an IVI of greater than 50. The most frequent species were the bigscale goatfish, *Pseudupeneus grandisquamis*; speckled flounder, *Paralichthys woolmani*; Pacific red snapper, *Lutjanus peru*; and the Peruvian mojarra, *Diapterus peruvianus* (Table 3); these four species presented the greatest abundances of the study, with 398, 327, 213 and 126 individuals, respectively.

**Table 3:** Abundance per station of fish species with highest IVI.

Species	S1	S2	S3	S4	S5	S6	S7
*C. orqueta*	1	2	1	3	6		3
*D. peruvianus*	13	9	3	15	49	26	11
*D. macropoma*	8	6			3	23	
*F. corneta*	1	5		2	2		1
*L. peru*	21	61	11	91	8	7	14
*P. woolmani*	24	24	44	22	36	74	96
*P. panamensis*	7	9	3	2	13	34	
*P. horrens*	1	6	2	1			2
*P. grandisquamis*	67	31	19	36	12	36	197
*S. pachygaster*	6	5	36	6		17	3
*S. scituliceps*	2	2			6	17	5

From the 12 fish species with higher IVI, only *P. grandisquamis* (IVI = 128.15), *P. woolmani* (IVI = 122.63), *L. peru* (IVI = 115.06) and *D. peruvianus* (IVI = 108.91) were present at all stations (Table 3), with maximum abundances per station of 197, 96, 91 and 49 individuals, respectively. The remainder of the species were absent from at least one station, with an average abundance of among two and 10 individuals per station.

**Figure 2:**
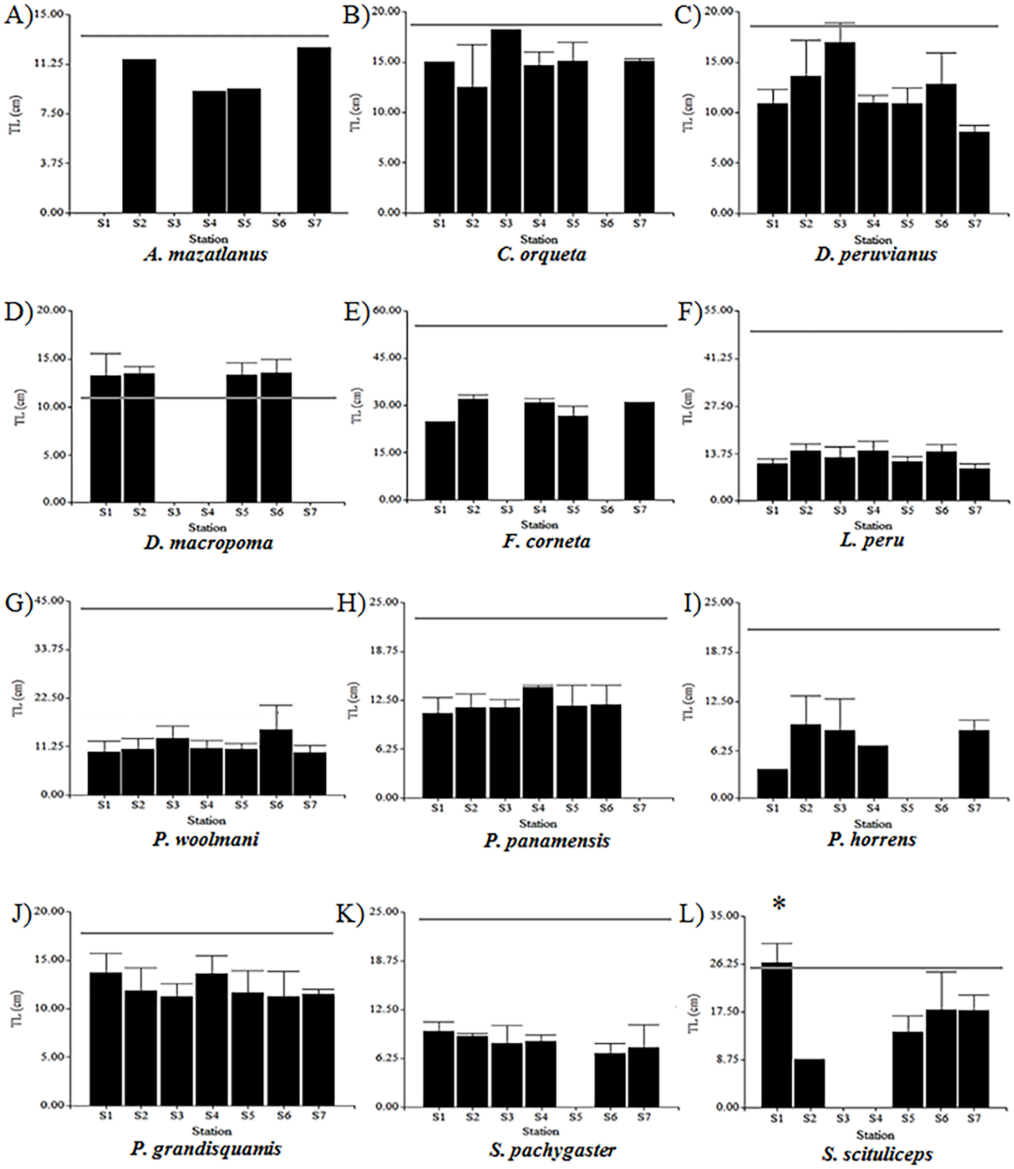
Fishes size structure at each sampling station. (A) *A. mazatlanus*. (B) *C. orqueta*. (C) *D. peruvianus*. (D) *D. macropoma*. (E) *F. corneta*. (F) *L. peru*. (G) *P. woolmani*. (H) *P. panamensis*. (I) *P. horrens*. (J) *P. grandisquamis*. (K) *S. pachygaster*. (L) *S. scituliceps*. The bars represent the average total length ± standard deviation. The gray line indicates the calculated length of maturity. ∗Represents significant differences in total length from each species among different sampled stations (*p* < 0.01).

No significant differences were found in the TL size structure in the majority of species according to latitudinal distribution; only *Synodus scituliceps* showed highly significant differences (*p* < 0.01), which were found in station 1 relative to station 2 (Fig. 2). The average sizes from *D. peruvianus*, *L. peru*, *P. woolmani* and *P. grandisquamis* were 12 cm (±2.77 cm), 12.71 cm (±2.81 cm), 11.51 cm (±3.17 cm) and 12.11 cm (±2.17 cm), respectively.

### Fish sex proportion and sexual maturity

**Figure 3:**
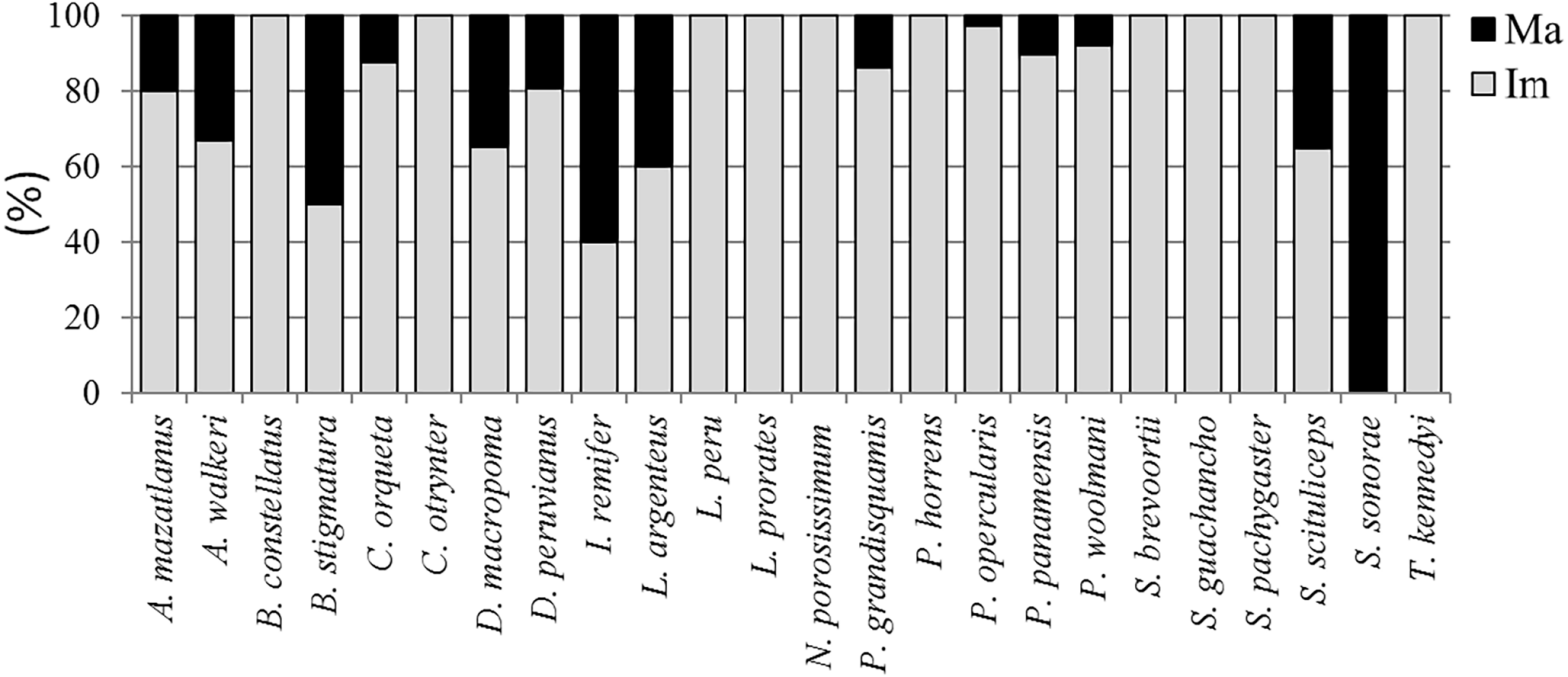
Juvenile and mature fish’s proportion. Ma, mature (developed gonad); Im, immature (undeveloped gonad). Bars represent 100% of organisms. Gray bar, percentage of immature individuals; Black bar, percentage of mature individuals.

**Figure 4:**
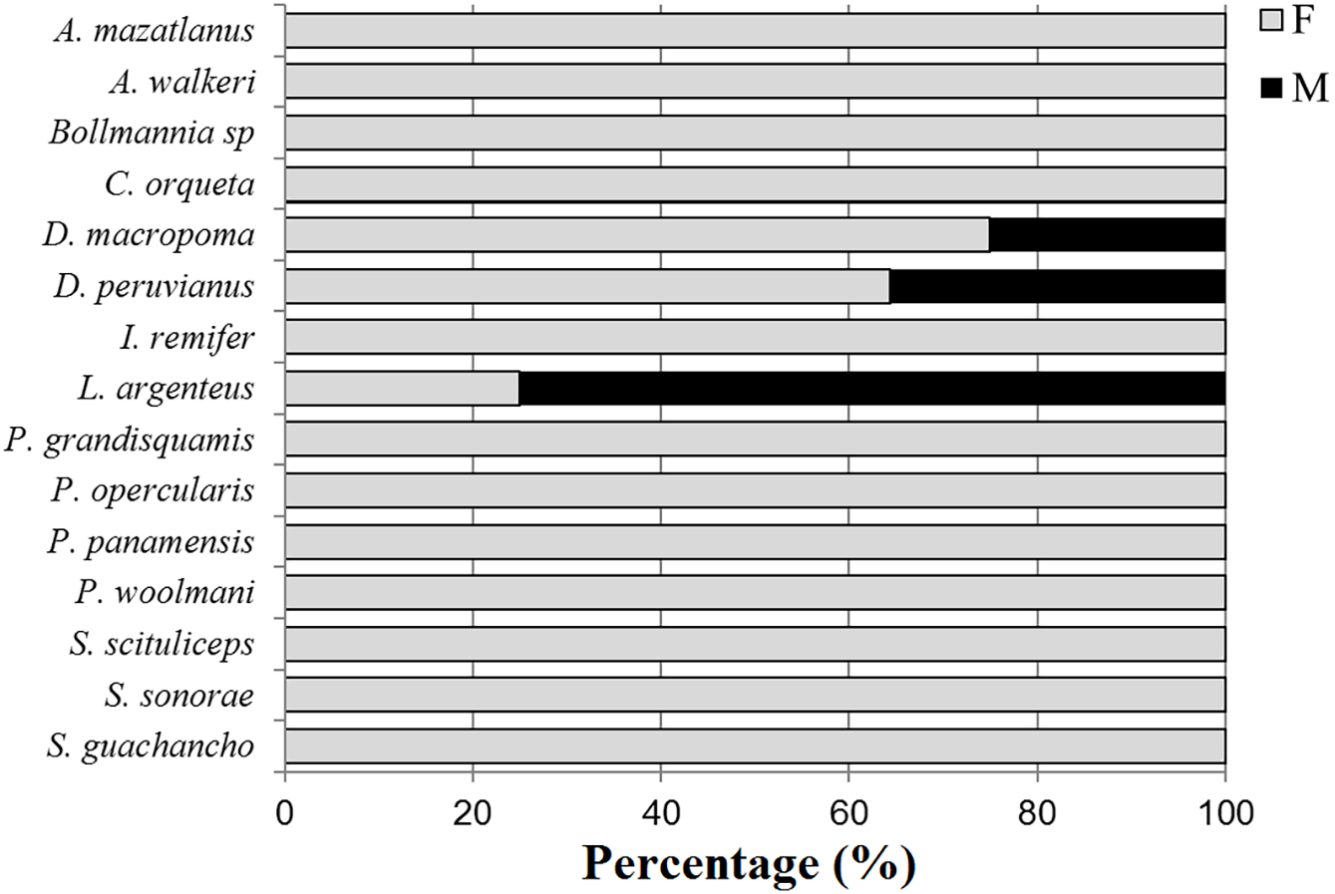
Sex proportion of fishes from shrimp bycatch. F, female; M, male. Bars represent 100% of organisms. Gray bar, percentage of females; Black bar, percentage of males.

The *TL*_*m*_ for the species with the highest IVI values were 18.5 cm for both *P. grandisquamis* and *D. peruvianus*, 44 cm for *P. woolmani* and 51.2 cm for *L. peru* (Table 2; Fig. 2). For all analysed species, fewer than 10% of fish presented developed gonads (Fig. 3); only the species *Scorpaena sonorae* presented 100% mature individuals (although individuals from this species were only found at station 6). The absence of developed gonads did not allow for sex determination from 23 of the 37 fish species analysed. From the remaining 14 species, 90% of the organisms were found to be female, and males were found in only three species: *Diplectrum macropoma*, *D. peruvianus* and *Larimus argenteus*. In this last species, the proportion of males (75%) was higher than females (Fig. 4).

## Discussion

The shrimp-bycatch in Mexico, as in other tropical countries, is composed of a wide diversity of molluscs, echinoderms, crustaceans and fish species (Herrera-Valdivia, López-Martínez & Castillo-Vargasmachuca, 2015), which are located principally in the nation’s coastal areas (Lucano-Ramírez et al., 2001). The present study is the first to analyse the sizes at first maturity of the fish species present in shrimp-bycatch (using both inferring and direct methods) as well as community structure. The diversity values from this work correspond to other works from the Gulf of California and other subtropical areas (Barreto, Polo & Mancilla, 2001; Mora, Jurado & Mendívez, 2010; Herrera-Valdivia, López-Martínez & Castillo-Vargasmachuca, 2015). The diversity index helps to estimate the health of an ecosystem (Jennings & Reynolds, 2000). It is important to note that the diversity found in this study only corresponds to a subsample of shrimp-bycatch fauna, all of which are discarded as waste.

Although any variation in the trophic chain (i.e., the interrelations among prey-predator species) could play a primary role in the distribution and abundance of each species, these kinds of studies are scarce due to the generally low level of interest in less charismatic species. Shrimping vessels do not consider variables such as temperature, dissolved oxygen, salinity and substrate, among others, and it does not seem feasible to establish these variables for better management strategies (Foster & Arreguin-Sánchez, 2014). This situation is mostly due to differences in the biometrics, behaviour, physiology and life history of each species (Loverich, 1995; Clucas, 1997; Eayrs, 2007). Most importantly, no single management/measurement solution fits all species (Foster & Arreguin-Sánchez, 2014).

In this study, the species richness, the similitude among stations and the size structure were not significantly found to be affected by the latitude or the month of sampling. As proof of this finding, the stations with the highest similitude were stations 1 and 7, which suggests a minimum effect of temperature on these variables. Temperature-related changes in distribution and abundance could be seen in northerly latitudes, where greater changes in temperature are generally found (Foster & Arreguin-Sánchez, 2014). Previous reports have established the sea bottom as one of the major determinants of the abundance of each species (Torres & Vargas, 2007; Nieto-Navarro et al., 2013). The species of fish found within shrimp-bycatch are typical of the sandy substrates of lagoon-estuarine systems where shrimp fishing is usually carried out (Rábago-Quiroz et al., 2011), with the exception of species associated with rocky and coral environments, such as *Chaetodon humeralis* and *Balistes polylepis*, both of which are considered low frequent.

The most representative families found in this study—Sciaenidae, Tetraodontidae, Haemulidae and Paralichthyidae, among others—are typical catches from tropical regions (Gibinkumar et al., 2012). Only 38% of these species were considered abundant and frequent, this suggests that most species were caught during the hauling or lifting of the nets, which also led to the low similitude among some of the sampled stations. The great majority of analysed species corresponded to benthopelagic species (*Caranx otrynter*, *Chloroscombrus orqueta*, *S. pachygaster* and *Isopisthus remifer*) and pelagic-neritic species (*Fistularia corneta*, *L. argenteus* and *Opisthonema libertate*), which coincides with the findings of previous reports from the Gulf of California and the Mexican Pacific (López-Martínez et al., 2010; Nieto-Navarro et al., 2013; González-Sansón et al., 2014).

The dominant species of the study—those with an IVI greater than 50—contributed more than 92% of the total abundance, which is typical of catches from the Gulf of California, Mexican Pacific and east Pacific (Allen & Robertson, 1994; González-Sansón et al., 2014; Rábago-Quiroz et al., 2011). Some studies have even reported that bycatch may be of greater volumes than those of the target fisheries (Barreto, Polo & Mancilla, 2001; Gibinkumar et al., 2012). The analysed organisms from previous studies have presented a large variety of size classes; most have been found to be juvenile organisms (Alverson et al., 1994; Liggins & Kennelly, 1996). A few authors have reported that shrimp-bycatch from the Gulf of California and the Mexican Pacific includes more than 100 small-size fish species (Perez-Mellado & Findley, 1985; Allen & Robertson, 1994; Alverson et al., 1994). For most fish species, no reports have been conducted to date on many of their biological aspects; such studies have only been conducted on the species *A. mazatlanus*, *L. peru*, *P. woolmani* and *P. grandisquamis* (Amezcua-Linares & Castillo-Rodriguez, 1992; Ramos-Santiago et al., 2006; Herrera-Valdivia, López-Martínez & Morales-Azpeitia, 2016; FishBase, 2017).

From the above discussion, we can see that it is necessary to seek strategies for the management and use of these species, since the vast majority of species have potential for commercialisation, and several of these species even have their own fisheries (FAO, 2017). One of the three fisheries indicators is the percentage of mature fish in a catch, which is an especially useful value for assessing eventual risks in fish stocks (Froese, 2004). In the literature, information on sexual maturity comes in various categories, including concepts (Froese & Pauly, 2000), symbols and definitions (Ragonese & Bianchini, 2014), all of which are closely related. The mean length at which fish of a given population become sexually mature for the first time (*L*_*m*_) is an important management parameter that is used to monitor whether a sufficient number of juveniles in an exploited stock can mature and spawn (Beverton & Holt, 1959; Ault, Bohnsack & Meester, 1998; Jennings, Reynolds & Mills, 1998).

For teleosts, Froese & Binohlan (2000) have examined the use of maximum length to predict the length at first maturity (Foster & Vincent, 2004) because their asymptotic length is highly correlated with maximum length. Froese & Binohlan (2000) also found the correlation among asymptotic length and first maturity length to be 85–91% across 265 bony fish species. For teleosts, the size at first maturity is estimated as the size at which 50% of the organisms have reached sexual maturity, the size at which ovaries appear (Kanou & Kohno, 2001), the size of the smallest recorded female with hydrated eggs (Nguyen & Do, 1996), the size of the smallest recorded first breeding, and the minimum size of a recorded female to release her eggs (Froese, 2004).

In this regard, it is important to emphasise that both first-maturity sizes and reproductive periods are not static but are subject to environmental variations and various analytical methods (Rodríguez-Domínguez et al., 2015). Even overfishing plays a leading role in population reductions by increasing food availability and growth rates, both of which may affect the size at first maturity (Froese & Binohlan, 2000). Furthermore, in adverse environmental conditions, some species tend to undergo gonadal resorption (Babin, 1987; Thomé et al., 2009), which is why the sex proportion and the size at first maturity are often underestimated. The present study analysed the sizes at first maturity of fish species present in shrimp-bycatch, of which more than 90% were found to be outside the limits of sustainable fisheries, that is, well below the size of first maturity.

Although some species have well-established fisheries, such as *L. peru* and *P. woolmani*, such species have no official regulations for minimum catch sizes; a minimum catch size has only been proposed for *L. peru*, at 31 cm TL (Ramos-Cruz, 2001; COFEMER, 2017). Foster & Vincent (2010) reported among several fish species from the Gulf of California an increase in reproductive activity from winter to spring, with possible peaks in summer, which coincides with the seasonal closing of the shrimp fishery. But the excessive capturing of juveniles in the spring will have a negative effect on that summer’s reproductive period and could result in more rapid depletion of small fish populations. Differences in sex proportion also indicate asynchronous maturation periods. The above evidence all demonstrates the problem of using generalised management strategies.

The National Commission of Aquaculture and Fisheries (Comision Nacional de Acuacultura y Pesca: CONAPESCA) is the Mexican governmental institution responsible for monitoring all shrimp vessels, both industrial and riverine, and enforces compliance. However, the primary problem is that trawling is conducted in shallow waters near the coast (Kelleher, 2004; Nieto-Navarro et al., 2013), which is where most species’ spawning and nesting areas are located (Lucano-Ramírez et al., 2001). On the other hand, since 2013 all Mexican shrimp vessels must have a fish excluder in their nets (Norma Oficial Mexicana, 2013); however, the abundance of fish species in shrimp-bycatch is still alarming. None of the analysed species in the current study, however, indicated a ‘hazard’ status or were threatened according to various conservation standards (Norma Oficial Mexicana, 2010; COFEMER, 2017; CONABIO, 2017), which may have been due to the scarcity of population and reproductive studies.

The inferring size at first maturity works as a first approach for creating management regulations until the specific data for each species becomes available (Froese & Binohlan, 2000). This study shows that many of the species present in shrimp-bycatch are at imminent risk because of their small sizes at catch and the large percentage of immature organisms within the catches; this situation not only affects the populations themselves but also causes damage to the whole trophic chain.

## Conclusions

The shrimp fishing industry is one of the most important global industries. This study, using data from northeast and southeast Mexican Pacific, the shrimp fishery found an impact on a total of 37 fish species, with an average of 15 species per sampled station, which negatively affects marine diversity. Diversity from fish species from shrimp-bycatch was found to be medium-low. There were no significant differences in specific richness and abundance of individuals among latitude and sampling month.

The dominant species were *P. grandisquamis*, *P. woolmani*, *L. peru* and *D. peruvianus*, with an IVI greater than 100 and a *TL*_*m*_ of 18.5, 43.9, 51.2, and 18.5 cm, respectively. The shrimp fishery was found to have a negative impact on reproduction of at least 12 fish species. Of the analysed fish, only 10% had developed gonads, of which 93% were found to be female. This work highlights the potential impact of the shrimp fishery on fish population dynamics and the need to improve the selectivity of shrimp trawls, and the imminent risk that marine communities face.

##  Supplemental Information

10.7717/peerj.4460/supp-1 Data S1 Raw data form diversity, abundance, frequency and length from fish from shrimp by catch at each station Diversity sheet shows the global data, for all species and stations. Besides relative density, relative frequency and IVI calculations. Stations sheets shows the species presence/absence, as well the abundance, total length, weight and maturity of each specie at that station. Graphics sheet summarizes the female/male proportion and the mature/immature proportion for each species. Froese and Binohlan sheet shows the calculation of Lm from the maximum length from FishBase.org.

10.7717/peerj.4460/supp-2 Data S2 Raw data form fish species with greatest IVI Each sheet shows one species; its abundance, frequency, length and weight data, as well as its gonadal development.

## Additional Information and Declarations

### Data Availability

The following information was supplied regarding data availability: Jimenez-Gutierrez, Rebeca (2018): Data S1. figshare. https://doi.org/10.6084/m9.figshare.5731185.v1. Jimenez-Gutierrez, Rebeca (2018): Data S2. figshare. https://doi.org/10.6084/m9.figshare.5746062.
